# Comparative genomics analysis of WAK/WAKL family in Rosaceae identify candidate WAKs involved in the resistance to *Botrytis cinerea*

**DOI:** 10.1186/s12864-023-09371-9

**Published:** 2023-06-19

**Authors:** Zicheng Wang, Yuan Ma, Meng Chen, Lingling Da, Zhen Su, Zhao Zhang, Xintong Liu

**Affiliations:** 1 Beijing Key Laboratory of Development and Quality Control of Ornamental Crops, Department of Ornamental Horticulture, College of Horticulture, China Agricultural University, Beijing, 100193 China; 2 State Key Laboratory of Plant Physiology and Biochemistry, College of Biological Sciences, China Agricultural University, Beijing, 100193 China; 3 Key Laboratory for Quality Regulation of Tropical Horticultural Crops of Hainan Province, School of Horticulture, Hainan University, Haikou, China

**Keywords:** Wall associated kinase, Rosaceae, Gene family, Comparative analysis, Gray mold

## Abstract

**Background:**

Wall associated kinase (WAK) and WAK-like (WAKL) are typical pattern recognition receptors act as the first sentry of plant defense. But little of WAK/WAKL family is known in Rosaceae.

**Results:**

In this study, 131 WAK/WAKL genes from apple, peach and strawberry were identified using a bioinformatics approach. Together with 68 RcWAK/RcWAKL in rose, we performed a comparative analysis of 199 WAK/WAKL in four Rosaceae crops. The phylogenetic analysis divided all the WAK/WAKL into five clades. Among them, the *cis*-elements of Clade II and Clade V promoters were enriched in jasmonic acid (JA) signaling and abiotic stress, respectively. And this can also be verified by the rose transcriptome responding to different hormone treatments. WAK/WAKL families have experienced a considerable proportion of purifying selection during evolution, but still 26 amino acid sites evolved under positive selection, which focused on extracellular conserved domains. WAK/WAKL genes presented collinearity relationship within and between crops, throughout four crops we mined four orthologous groups (OGs). The WAK/WAKL genes in OG1 and OG4 were speculated to involve in plant-*Botrytis cinerea* interaction, which were validated in rose via VIGS as well as strawberry by qRT-PCR.

**Conclusions:**

These results not only provide genetic resources and valuable information for the evolutionary relationship of WAK/WAKL gene family, but also offer a reference for future in-depth studies of Rosaceae WAK/WAKL genes.

**Supplementary Information:**

The online version contains supplementary material available at 10.1186/s12864-023-09371-9.

## Background

In the process of growth and development, plant must respond rapidly to environmental changes and various biotic and abiotic stresses [1]. Receptor-like kinases (RLKs) are a class of protein that widely located on the surface of plant cell and usually contain extracellular signal-sensing and intracellular kinase structures. RLKs play an important role in transducing extracellular signals by perceiving changes of polysaccharides, proteins, lipids and other ligands [2, 3]. RLKs are ubiquitous in plant species, including no less than 17 subfamilies [4, 5]. Wall associated kinase (WAK) and WAK-like (WAKL) is one subfamily of RLKs [6].

With at least one transmembrane domain, WAK/WAKL proteins have an intracellular domain of serine/threonine kinase (PKinase) at the C-terminus and complex N-terminal extracellular domains, including calcium-binding EGF domain (EGF_CA) and galacturonic binding domain (GUB_WAK_bind). EGF_CA is composed by about 40 residues of epidermal growth factor (EGF). The N-terminal of EGF protein is often accompanied by a calcium-binding site, and calcium is required for their biological functions [7]. The cysteine-rich GUB_WAK_bind domain is a characteristic structure of WAK/WAKL, which interacts with homogalacturonans, the pectin fragment of plant cell wall [8]. It has been shown that WAK/WAKL family plays an important role in effective intracellular and extracellular communication, regulating root growth [9], stem strength [10] and disease resistance [11, 12].

The polyphagous necrotrophic fungi *Botrytis cinerea* is one of the major pathogens causing gray mold disease, with 1400 known hosts covering 586 plant genera [13]. Global economic losses caused by *B. cinerea* can reach hundred-billions of dollars annually [14]. *B. cinerea* has been listed as the second largest plant disease in the world [15], and is also the main postharvest disease of rosaceous crops (such as strawberries and roses) [16]. Arabidopsis AtWAK1 was the first reported recognition receptor for *B. cinerea* [17].

Rosaceae crops, especially apple, peach, strawberry and rose, are extremely susceptible to *B. cinerea*, however the systematic comparative analysis of WAK/WAKL genes in Rosaceae has not been reported. In this study, we compared the structure, phylogenetic relationships, selection pressure and collinearity relationships of the WAK/WAKL gene family in four Rosaceae crops, including apple (*Malus domestica*), peach (*Prunus persica*), strawberry (*Fragaria vesca*) and rose (*Rosa chinensis*). Our results will provide useful information and theoretical support for further study of the WAK/WAKL biological functions in Rosaceae.

## Materials and methods

### Identification of the WAK/WAKL genes in Rosaceae crops

The information of Rosaceae genome were obtained from Genome Database for Rosaceae, containing gddh13 v1.1 for Apple (*Malus x domestica*) [18], Fragaria vesca v4.0.a1 for strawberry (*Fragaria vesca*) [19] and Prunus persica v2.0 for peach (*Prunus persica*) [20, 21]. Firstly, to identify the nonredundant WAK genes, the HMM files (EGF_ CA, PF07645.18; GUB_WAK_ bind, PF13947.9; PKinase_Tyr_ser, PF07714.20) generated from protein families (Pfam) website were used for hmmsearch with *e*-value < 1e^− 3^. All candidate WAK/WAKL members were validated transmembrane helix (TMhelix) by TMHMM-2.0 and signal peptide (SignalP) by SignalP-5.0 (https://services.healthtech.dtu.dk). Finally, we selected the WAK/WAKL members with SignalP, EGF_CA or GUB_WAK_bind, TMhelix and PKinase.

### Phylogenetic analysis of the WAK/WAKL in Rosaceae

WAKs/WAKLs protein sequences were aligned by algorithm ClustalW in MGEA7 with default parameters [22]. Then use neighbor joining (JFF + G model) with 1000 bootstrap replicates to construct the phylogenetic tree.

Domain position of Pkinase, GUB_WAK_Bind and EGF_CA was generated from conserved domain database (CDD) (https://www.ncbi.nlm.nih.gov/cdd) and Pfam (http://pfam-legacy.xfam.org) online predicting and removing redundant results before visualization, TMhelix and SignalP are directly based on the online prediction outputs of Technical University of Denmark, and gene structure is extracted from GFF/GTF files from their respect genomes. Finally, the amino acid sites of the domain were used as input files in TBtools [23]. For amino acid sequence logo exhibition, WebLogo3 online application was performed [24].

### Selective pressure analysis

PAML4.9j was used for selective pressure analysis by branch model and site model [25]. The branch model considers the ω (dN/dS) value represented the adaptive evolution between every end branch in phylogenetic tree. Site model assumes various selection pressures at different codons. M0/M3 is used to detect the consistency of ω-ratios between sites. The positively selected sites were identified by M1a/M2a and M7/M8. When positive selection is detected, M2a and M8 can be used to further identify the amino acid sites via Bayes Empirical Bayes (BEB) algorithm.

### Promoter region analysis of the WAK/WAK in Rosaceae

2000 bp promoter sequences of Rosaceae WAK/WAKL were extracted from DNA sequence before translation initiation codon (ATG). *Cis*-elements were forecasted on PlantCARE (http://bioinformatics.psb.ugent.be/webtools/plantcare/html/). Conventional elements such as: short function, core promoter element around − 30 of transcription start, common *cis*-element in promoter and enhancer regions were deleted. Other elements in the output file were manually sorted and simplified based on previous classification rules [26–28] (Supplementary Table 1).

### Heat map analysis

RNA-seq data were obtained from rose petals treated with a variety of plant hormones [29], including abscisic acid (ABA) treatment, auxin (2,4D), cytokinin (6-BA), gibberellin (GA3), jasmonic acid (JA) and salicylic acid (SA). The fragments per kilobase per million reads (FPKM) values which measure gene expression abundance were converted by min-max normalization between different hormone treatments to insulate the heat maps from extreme values.

### The paralogous and orthologous analysis of WAK/WAKL in Rosaceae

Selection of paralogous genes and orthologous genes share a common set of parameters, determination of collinearity is proceeded by MCscanX [30] with default parameters. Selecting collinear blocks are conducted according to the following parameters: match score: 50, match size: 5, overlap window: 5, *e*-value: 1e and max gaps: 25. The orthologous groups are selected manually through the MCscanX output file, and the visualization is displayed by TBtools software.

### Virus induced gene silencing (VIGS)

A 249 bp specific fragment of *RcWAK8* was cloned from rose petal cDNA and finally inserted to TRV2 vector (TRV2-RcWAK8) [31]. Then, take down the outer petals of rose at stage 2 of flower opening [32], and make a 12.5-mm diameter petal disc with a hole punch in the middle of the petals. *Agrobacterium tumefaciens* containing TRV1 and recombinant TRV2 vector were mixed in a ratio of 1:1, and vacuum suction was used to make agrobacterium invade plant tissues. To ensure a stable phenotype, the VIGS assay was repeated at least three times with at least 48 petals each time.

All the constructs and plant materials used in this study are freely available from the corresponding author, for research purposes. There no special permissions are necessary to collect item.

### *B. cinerea* inoculation and qRT-PCR analysis

The method for *B. cinerea* cultivation has been described previously [33]. Briefly, the *B. cinerea* strain B05.10 for strawberry and rose petal discs was grown on potato dextrose agar (PDA) at room temperature for two weeks. The spores were harvesting with deionized water and then suspending in potato dextrose broth (PDB) to a final concentration of 1 × 10^5^ conidia/mL.

For strawberry (*Fragaria vesca*) inoculation, 5-µL drops of *B. cinerea* inoculum or PDB (mock) were dropped onto the ripe fruits, and the fruits were placed on petri dishes with wet filter paper to ensure 100% humidity. All the samples were harvested at 48 h post inoculation (hpi) with four biological repeats and the fruits were immediately frozen in liquid nitrogen and stored at -80 °C.

For detached rose petal discs, six days after recombinant tobacco rattle virus (TRV) vector’s infection, petal discs were inoculated with 2 µL spore suspension of *B. cinerea*. All the petal disc were placed on petri dishes with 0.4% agar (v/v) to ensure 100% humidity. All samples were photographed at 60 hpi, and the lesion area was measured using the ImageJ. Finally, they were immediately frozen in liquid nitrogen and stored at -80 °C.

RNA extraction was carried out with the E.Z.N.A Plant RNA Kit (OMEGA), reference plant RNA difficult sample protocol. cDNA was generated using Takara Reverse Transcriptase M-MLV, and 1 µL of the first strand cDNA was used as a template in the reaction with the KAPATM SYBRR quantitative PCR kit (Takara), which was run on a StepOnePlus Real-Time PCR System (Thermo Fisher Scientific). FvACTIN and RcUBI was used as a housekeeping gene. The primers used for determining transcript abundance are listed in Supplementary Table 2.

All the study protocols must comply with relevant institutional, national, and international guidelines and legislation.

## Results

### Identification and classification of Rosaceae WAK/WAKL gene family

**Fig. 1:**
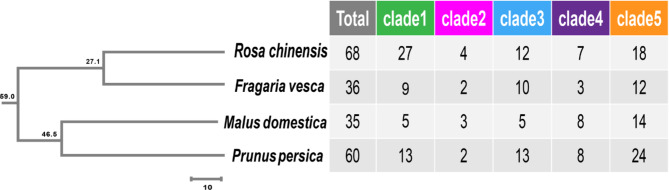
The phylogenetic tree of Rosaceae species. The phylogenetic evolutionary tree indicates the order of divergence of the species, and the number on the node indicates the time of divergence (million years ago, MYA).

As previously stated, PKinase (PF07714.20), GUB_WAK_bind (PF13947.9), EGF_CA (PF07645.18) are typical domains for WAK/WAKL members, in addition signal peptide (signalP) and transmembrane helix (TMhelix) were necessary. The genes contained three typical domains were named WAK, the ones without GUB_WAK_bind or EGF_CA were named WAKL. Based on these rules, 68 *RcWAK/RcWAKL* family members have been identified in rose (*Rosa chinensis*) [34]. In this study, a total of 131 non-redundant Rosaceae *WAK/WAKL* genes were identified (Fig. 1), including 36 in strawberry (14 *FvWAKs*, 22 *FvWAKLs*), 35 in apple (9 *MdWAKs*, 26 *MdWAKLs*), 60 in peach (12 *PpWAKs*, 48 *PpWAKLs*) (Supplementary Fig. 1). Their gene name, accession number, the number of introns and exons, the length of coding sequence (CDS) and amino acid sequence, as well as the chromosome location were shown in the Supplementary Table 3 ~ 5. All *WAK/WAK*L genes were named according to their order on the chromosomes. Among them, *PpWAKL5* had the shortest CDS coding 408 aa, and *FvWAK12* was the longest one coding 2266 aa. The mean of CDS length was 2341 bp and the average amino acid sequence length was 717 aa among all 131 WAK/WAKL.

In summary, a total of 199 Rosaceae *WAK/WAKL* were obtained from four Rosaceae crops. Comparative analysis of *WAK/WAKL* family members in strawberry, apple, peach and rose showed that the longest average length of the *WAK/WAKL* CDS was 2973 bp in strawberry, followed by apple, pear and rose. The average sequence length of *WAK/WAKL* in apple (2194 bp), rose (2036 bp) and peach (2047 bp) is similar, but the length in strawberry is 32.9% larger. Meanwhile, the *WAK/WAKLs* were different distributions on chromosomes in four Rosaceae crops. The *FvWAK/FvWAKL* and *RcWAK/RcWAKL* are distributed on all the 7 chromosomes in strawberry and rose. However, there is no *MdWAK/MdWAKL* gene on chromosome 3, 11 and 16 among 17 chromosomes of apple and no *PpWAK/PpWAKL* gene on chromosome 7 and 8 among 8 chromosomes of peach. In addition, the *WAK/WAKL* were unevenly distributed across each Rosaceae specie chromosomes, e.g.: the high density of *PpWAKs/PpWAKLs* location was observed at 19.63 ~ 19.84 Mb on chromosome 3 (*PpWAKL23* ~ *PpWAKL33*) and at 4.59 ~ 4.77 Mb on chromosome 4 (*PpWAK2* ~ *PpWAK12*). The unbalanced distribution of *WAK/WAKL* genes indicated genetic variation of Rosaceae crops during the evolutionary process.

### Phylogenetic analysis of WAK/WAKL genes in four Rosaceae crops

**Fig. 2:**
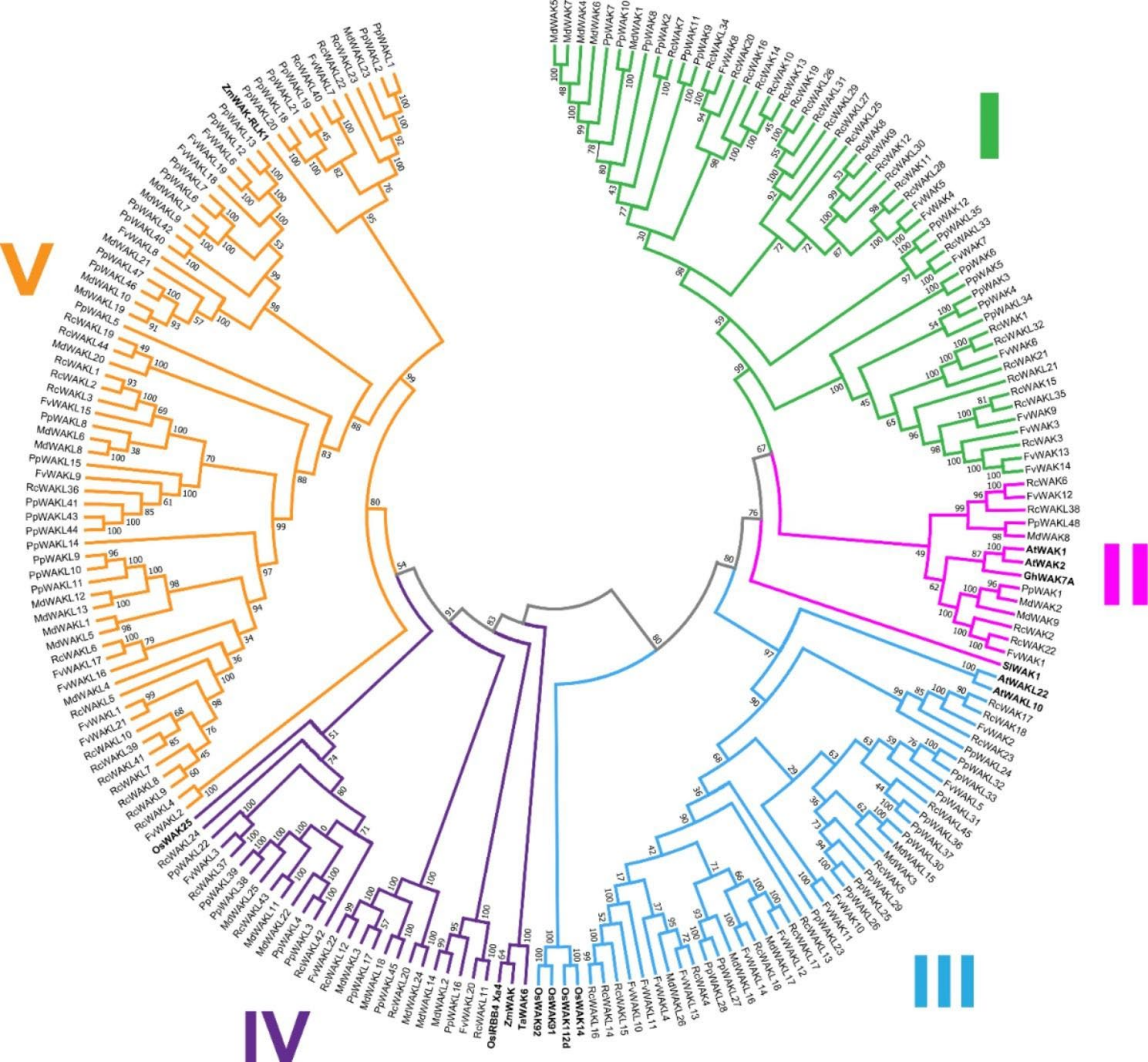
Phylogenetic analysis of the Rosaceae WAK/WAKL with defense-related WAK/WAKL from other plant species. Complete alignments of the Rosaceae and the defense-related WAKs/WAKLs from other plant species, including Arabidopsis, cotton (*Gossypium hirsutum*), rice (*Oryza sativa*), tomato (*Solanum lycopersicum*), maize (*Zea mays*), and wheat (*Triticum aestivum*), were used to construct a phylogenetic tree using the Neighbor-Joining method. The bootstrap values are indicated on the nodes of the branches. The WAKs/WAKLs reported to be involved in plant disease resistance are marked in bold. Group I-V were labled in green, magenta, blue, purple and orange, respectively

In order to explore the evolutionary relationship among 199 WAK/WAKL in Rosaceae crops, we constructed a rootless phylogenetic tree using the Neighbor-Joining algorithm, included resistance related WAK/WAKL genes AtWAK1, AtWAK2, AtWAKL10, AtWAKL22, GhWAK7A, OsWAK14, OsWAK91, OsWAK92, OsWAK112d, OsWAK25, OsIRBB4_Xa4, SlWAK1, TaWAK6, ZmWAK, and ZmWAK-RLK1 [10, 12, 17, 35–40]. We divided all the WAK/WAKL genes into five clades [6, 26]. Clade V was the largest group contains 68 genes, accounting for 34.2% of all family genes. Followed by Clade I (54 genes), which was the only clade had no verified disease resistance genes. Clade II had the least number of genes containing only 11 genes (Figs. 1 and 2). The conserved domains and gene exon-intron structure were visualized on the basis of evolutionary analysis (Supplementary Fig. 2). Rosaceae WAKLs were intensively distributed in Clade IV and Clade V. Although we defined those genes lacking GUB_WAK_bind or EGF_CA as WAKLs, only 9 of 134 Rosaceae WAKLs actually lacked GUB_WAK_bind, while all the others were missing EGF_CA. The number of exons of Rosaceae WAK/WAKL varied greatly, from 1 to 12. There was also a big difference in Rosaceae WAK/WAKL gene length, the shortest one was *RcWAKL14* (1448 bp) and the longest one was *FvWAK12* (17,454 bp). Interestingly, the *FvWAKLs* and *MdWAKLs* in CladeV had more exon-intron structures, but *RcWAKLs* and *PpWAKLs* were not.

**Fig. 3:**
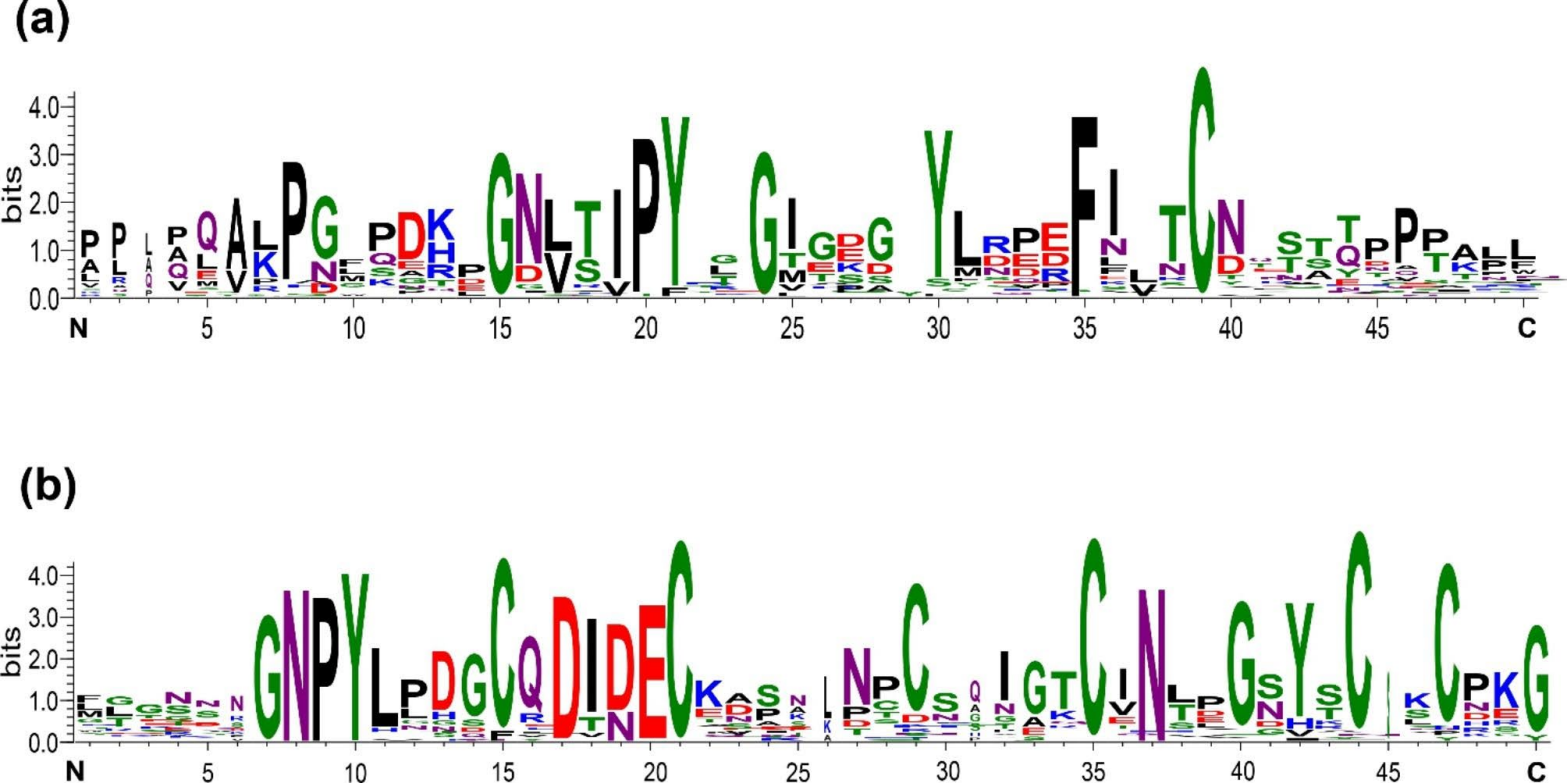
Amino acid sequences of WAK/WAKL characteristic domains in four Rosaceae crops. **a** Typical amino acid for galacturonan-binding domain. **b** Typical amino acid for calcium-binding EGF domain. the size of the letter indicates the probability of amino acid occurrence at this site after multiple alignments

After multiple sequence alignment, we clearly found two characteristic domains of WAK/WAKL in the alignment results (Fig. 3). EGF_CA with six conserved cysteines for calcium-binding and GUB_WAK_bind located near the N-terminal end of the extracellular domain.

### WAK/WAKL underwent purifying selection in four Rosaceae crops

The dN/dS (*ω*) nucleotide substitution ratios were used to evaluated the selection pressure among WAK/WAKL between different branches in phylogenetic tree. Generally, *ω* < 1 is consistent with purifying selection, while *ω* > 1 indicates a positive selection. With M0 model, which assumes that all branches have equal *ω* values, we got a *ω* = 0.254. With M1 model, which assumes that all branches have unequal *ω* values, 86.7% of *ω* values among different branches were < 1. All the results showed that WAK/WAKL genes undergone a strong purifying selection during their evolutionary history (Table 1).

**Table 1:** Parameter estimates and likelihood values tests for the branch models

Model	np^1^	Ln L^2^	Estimates of parameters	LRT^3^*P*-value
M1	140	-48290.16		< 0.01
M0	135	-50696.98	ω = 0.254	

np^1^: number of parameters;

Ln L^2^: logarithm of maximum likelihood;

LRT^3^: likelihood ratio test

We further use the site model to detect the sites of protein sequences which occurred positive selection (Table 2). The results showed that the M3 model with LRT *P*-value < 0.01 was better than the M0 model, indicating that different amino acid sites have different selection pressures in WAK/WAKL family. Further results demonstrated that a total of 26 amino acid sites were identified in the M2a and M8 models that were subject to positive selection. The positive sites were mostly located in the extracellular domains of WAK/WAKL family members, mainly concentrated on the GUB_WAK_bind domain.

**Table 2:** Parameter estimates and likelihood values tests for the site models

Model	np^1^	Ln L^2^	Estimates of parameters	LRT^3^ *P*-value	Positive sites
M3	401	-48739.81	p = 0.257,0.337,0.406 ω = 0.273,0.258,0.505	< 0.01	Not Found
M0	397	-50572.42	ω0 = 0.254		Not Allowed
M2a	400	-49852.17	p = 0.639,0.193,0.169	< 0.01	34T 41 H 43R 77G 99G 100E 102 H 115T 118D 140 V 142P 166 L 176Y 241 S 283 H
M1a	398	-49557.35	ω = 0.153,1.000,2.147 p = 0.639,0.361 ω = 0.153,1.000		Not Allowed
M8	400	-48365.71	p0 = 0.983, p = 0.634, p1 = 0.016, q = 1.870, ω = 1.378	< 0.01	33 A 41 H 43R 57 S 69G 71 L 74E 99G 100E 108G 115T 118D 126D 129 S 134 S 135P 138T 140 V 142P
M7	398	-47651.67	p = 0.627, q = 1.751		Not Allowed

np^1^: number of parameters;

Ln L^2^: logarithm of maximum likelihood;

LRT^3^: likelihood ratio test

### Promoter *cis*-elements analysis of *WAK/WAKL* genes in Rosaceae crops

To investigate the *cis*-elements in promoter sequences of *WAK/WAKL* genes, 2 kb of DNA sequences up-stream of the initiation codon (ATG) were analyzed by Plant_CARE. We classified the identified *cis*-elements into three major categories, including plant development and growth, phytohormone response and abiotic response (Supplementary Table 1). We speculated that *WAK/WAKL* genes in different clade played a role in different biological processes. The predicted *cis*-elements were analyzed according to different clades in phylogenetic tree (Table 3). The *cis*-elements responsive to abscisic acid (ABA) and associated with abiotic stress accounted for 25.52% in Clade I. The *cis*-elements in Clade II and Clade III prominently responded to MeJA response, accounting for 27.43% and 24.65%, respectively. Abiotic stress-responsive *cis*-elements are enriched in Clade IV and Clade V.

**Table 3:**
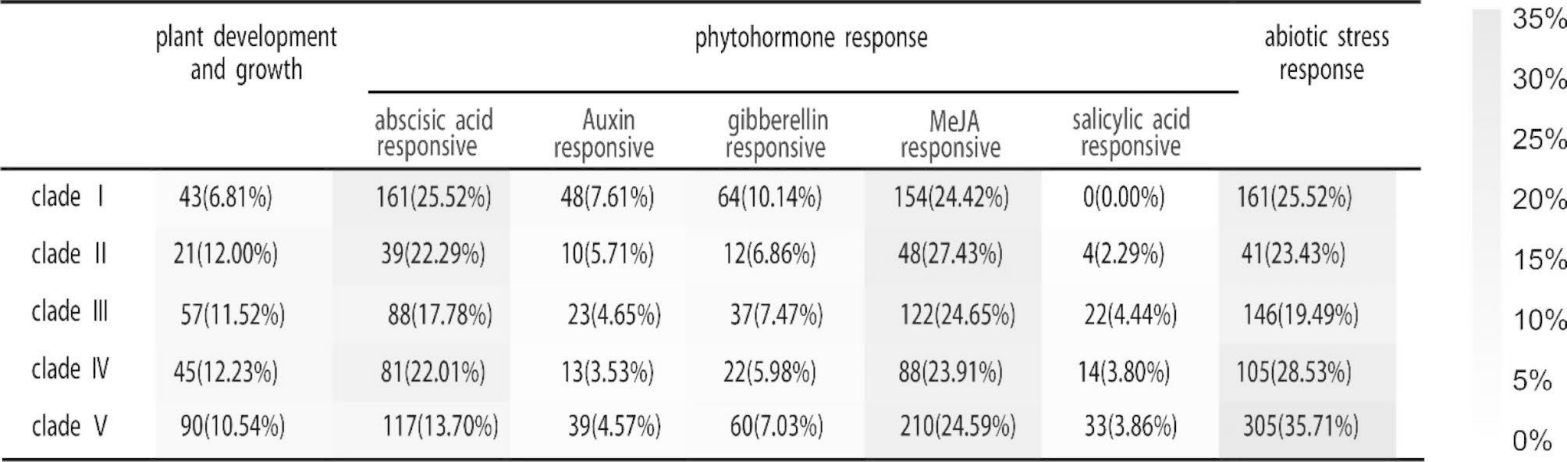
Promoter *cis*-elements enrichment analysis of Rosaceae WAK/WAKL

**Fig. 4:**
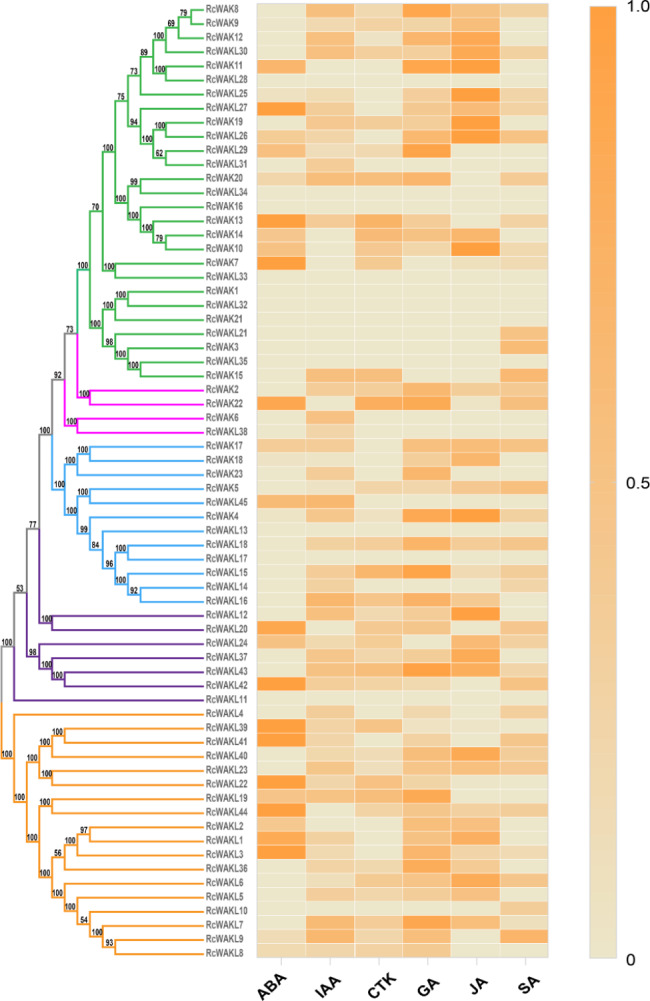
Expression analysis of WAK/WAKLs across different hormone treatment. FPKMs were normalized by min-max method to represent relative expression levels. Evolutionary tree on the left refers to the phylogenetic relationships in the identification of WAK/WAKL gene families in rose [34]. Group I-V were labeled in green, magenta, blue, purple and orange, respectively

In order to explore the expression patterns of WAK/WAKL family genes under different hormone treatments, we analyzed the transcriptome of rose flowers responding to different hormone treatments [29]. According to the heat map (Fig. 4), Clade I members showed strong response to ABA and JA, followed by GA. while members of Clade II did not show a more significant response to a particular hormone. Clade III members tend to respond to JA and GA3. And Clade IV and Clade V showed strong response to ABA, followed by JA and GA3. The transcript expression patterns of R*cWAK/RcWAKL* in hormone treated RNA-seq data was consistent with promoter *cis*-elements enrichment analysis of Rosaceae WAK/WAKL (Fig. 4; Table 3).

### The collinearity relationships of *WAK/WAKL* in four Rosaceae crops

To further investigated the *WAK/WAKL* evolutionary trajectory events, we have constructed paralogous gene pairs and orthologous gene groups in or between four Rosaceae crops, respectively. Collinearity analysis have identified 6, 2, 4 and 0 *WAK/WAKL* paralogous gene pairs in apple, peach, rose and strawberry, respectively (Supplementary Fig. 3). Interestingly, rose possessed four paralogous gene pairs, three of them were located on chromosome 5 (11.37 ~ 12.01 Mb), included *RcWAK7, RcWAK8, RcWAK9, RcWAK11, RcWAK13*, and *RcWAK14*. This result implied the small-scale tandem replication events within rose genome. In addition, apple with the largest genome size has the least *WAK/WAKL* number among four Rosaceae crops. Meanwhile, 45.7% (16/35) of *MdWAK/MdWAKL* were detected as paralogous gene. The results revealed that *MdWAK/MdWAKL* experienced high-pressure selection under evolutionary in apple, the expansion of *MdWAK/MdWAKL* family genes mainly depend on whole-genome duplication (WGD).

**Fig. 5:**
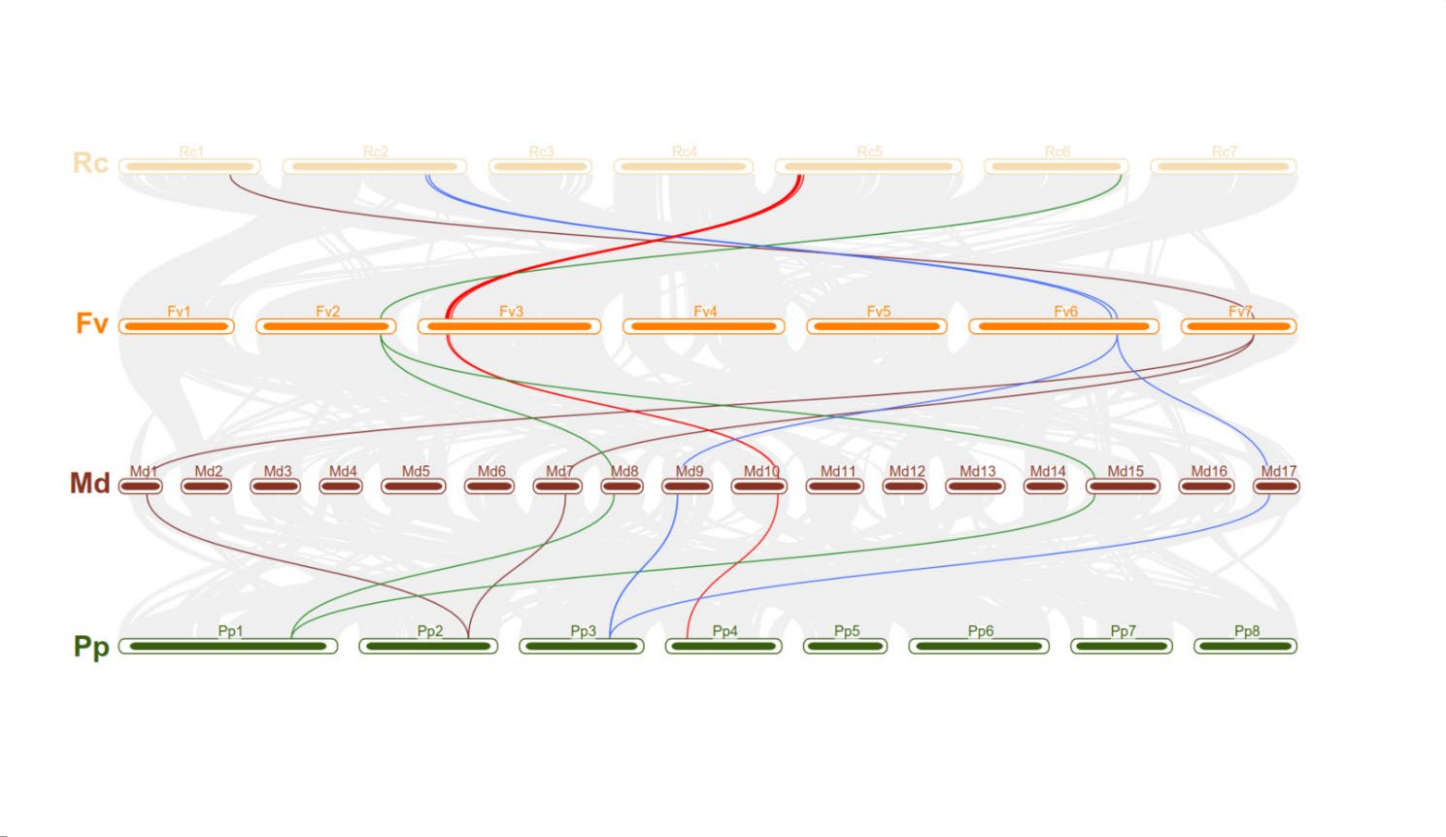
Four groups of orthologous genes running through Rosaceae species. We constructed a syntenic map to visualize the orthologous groups with red, blue, brown and green lines respectively

Orthologous group is used to describe a cluster of genes evolved from a single gene in the last common ancestor (LCA) among crops. Here, we constructed a syntenic map and compiled a gene list of *WAK/WAKL* orthologous genes across the four Rosaceae crops. 71 of 199 *WAK/WAKL* genes were mapped in orthologous blocks, accounting for 35.7% of all genes. They were divided into fourteen orthologous groups (Supplementary Fig. 4). There were four orthologous groups OG1, OG3, OG4 and OG6 were highly conserved across four Rosaceae crops (Fig. 5; Table 4). Although *WAK/WAKL* genes underwent different degrees of contraction and expansion during the divergence of the four species, the gene functions in same orthologous group may have the similar function.

**Table 4:** Orthologous gene group among four Rosaceae crops

Orthologous group NO.	rose	strawberry	apple	peach
**OG1**	RcWAK7	FvWAK2	MdWAK4	PpWAK7
	RcWAK8	FvWAK3	MdWAK6	
	RcWAK11	FvWAK5		
	RcWAK15	FvWAK7		
	RcWAKL25	FvWAK8		
	RcWAKL28	FvWAK9		
	RcWAKL29			
	RcWAKL33			
	RcWAKL34			
**OG2**	RcWAKL15	FvWAKL10	MdWAKL15	PpWAKL23
	RcWAKL17	FvWAKL12	MdWAKL26	PpWAKL25
**OG3**	RcWAKL11	FvWAKL20	MdWAKL2	PpWAKL16
			MdWAKL14	
**OG4**	RcWAK22	FvWAK1	MdWAK2	PpWAK1
			MdWAK9	

### The orthologous groups implied some Rosaceae WAKs playing important role in *B. cinerea* resistance

As the same orthologous group has shared ancestry, the genes in one group may have the similar gene function between crops. In our previous report, *RcWAK8* (OG1) and *RcWAK22* (OG4) were significantly up-regulated expression in rose petals during *B. cinerea* infection [34]. These results indicated the genes from OG1 and OG4 may involve in plant-pathogen interaction.

**Fig. 6:**
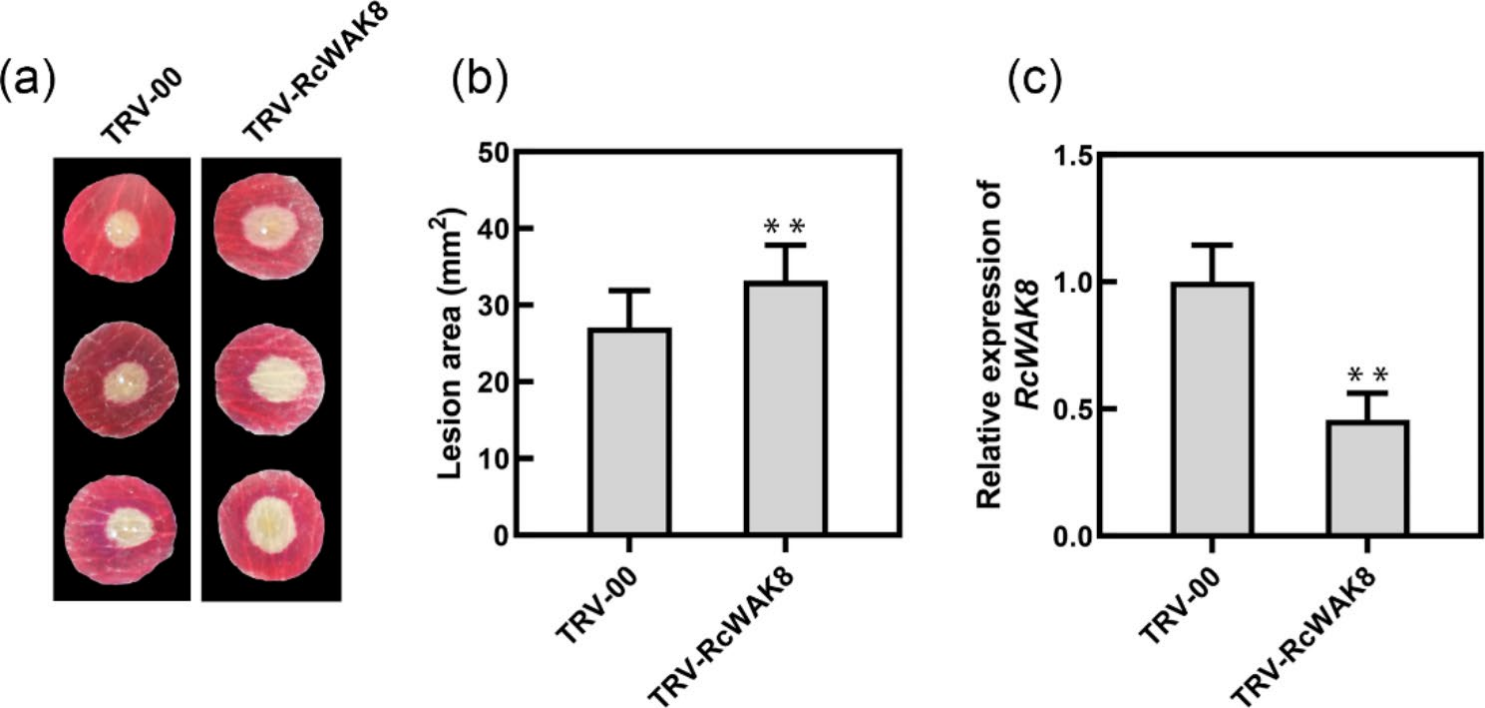
Gene Function Analysis of *RcWAK8* in Rose. (**a**) Compromised *B. cinerea* resistance symptoms on rose petal disks upon the silencing of *RcWAK8*, shown at 60 hpi (hours post inoculation). A recombinant tobacco rattle virus (TRV) targeting *RcWAK8* (TRV-RcWAK8) was used for the gene silencing, and an empty TRV (TRV-00) was used as the control. (**b**) Quantification of the average diameter of the disease lesions on the control and *RcWAK8*-silenced petals at 60 hpi. Error bars = standard deviation. The statistical analysis was performed using a Student’s *t*-test; ** *P* < 0.01. (**c**) Quantification of *RcWAK8* expression in TRV-RcWAK8-inoculated petal discs compared to that in the control.

To further verified the function of RcWAK8 and RcWAK22 in rose-Botrytis interaction, we attempt to knock down the expression of RcWAK8 and RcWAK22 one by one via VIGS. However, the VIGS must ensure the specificity of inserted fragments (without no consecutive 23 bp sequence is exactly the same) [41]. Unfortunately, the coding sequence of RcWAK22 is too similar to RcWAK2 (Supplementary Fig. 5). By contrast, blast analysis suggested TRV-RcWAK8 construct do not have any off-target silencing in rose, it is specifically targeting *RcWAK8*. Petal discs silenced with RcWAK8 gene showed significant lesion enlargement compared with controls (Fig. 6a and b). Meanwhile, the silencing efficiency was also confirmed by qRT-PCR (Fig. 6c). All these evidences suggest that *RcWAK8* plays an important role in the resistance of rose petals to gray mold.

**Fig. 7:**
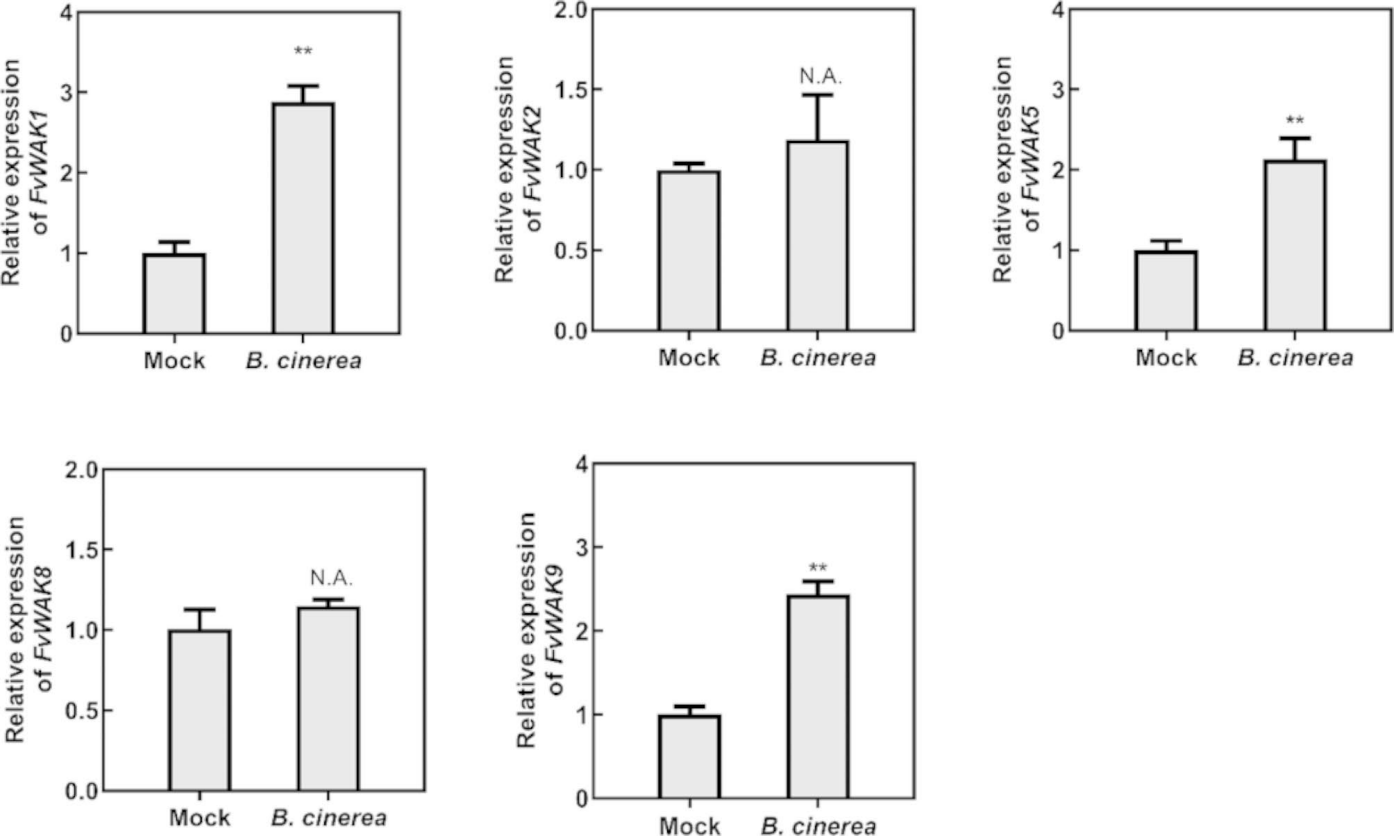
Validation of *FvWAK/FvWAKL* from orthologous groups. The friuts of strawberry were collected after B. cinerea inoculated 48 h. *FvACTIN* was used as a reference gene. The primers used for each *FvWAK/FvWAKL* were listed in Supplementary Table 5. The value represented the mean of three technical replicates ± SD. Experiments were performed independently 3 times, with similar results. Statistial analysis were performed by Student’s *t*-test (N.A, Insignificant, ** *P* < 0.01)

On the other hand, to verify expression patten of *FvWAK/FvWAKLs* during *B. cinerea* infection in OG1 and OG4, real-time quantitative PCR (RT-qPCR) was performed to test the expression of *FvWAK1, FvWAK2, FvWAK3, FvWAK5, FvWAK7, FvWAK8* and *FvWAK9* in strawberry fruits after *B. cinerea* infection 48 h. As shown in Fig. 7, the expression level of *FvWAK1* (OG4), *FvWAK5* (OG4) and *FvWAK9* (OG4) were significantly increased in *B. cinerea*-treated fruits comparing with mock (PDB-treated fruits). *FvWAK2* and *FvWAK8* have no change in *B. cinerea*-treated fruits. *FvWAK3* and *FvWAK7* were undetermined in all samples, may because these two *WAKs* existed tissue-specific expression.

## Discussion

WAK/WAKL family is one of the important receptor-like kinases (RLKs) localized on the cell membrane. Here, we use comparative genomics analysis of four Rosaceae WAK/WAKL family genes to provide novel insights in gene function and evolution. The four Rosaceae crops diverged around the 59 million years ago (MYA), corresponding to the Cretaceous-Paleogene (K-Pg) mass extinction event, with a large number of genome ploidy events occurring [42–44] (Fig. 1). It is implied that whole-genome duplication (WGD) events may have helped crops adapt to the harsh climatic conditions of the time, allowing them to survive the extinction event [45]. However, the number of *WAK/WAKL* family genes was insignificantly related with WGD event or the genome size (Supplementary Table 6). For example, apple with a genome of 742 Mb had a neoteric WGD event at about 30–45 MYA, but only 35 *WAK/WAKL* genes were identified, a relatively fewer number among crops (Fig. 1, Supplementary Table 6). MdWAK/MdWAKL family seems no expansion event happened during the WGD. It might be apple (‘Golden Delicious’) experienced a long period of artificial domestication selection after WGD, genes with redundant functions are streamlined.

The phylogenetic evolutionary tree of WAK/WAKL showed that RcWAK/RcWAKL and FvWAK/FvWAKL were classified on the closest terminal branch, as well as WAK/WAKL of peach and apple (Fig. 2), supporting the relationship of crops divergence (Fig. 1). Meanwhile, we clearly found two characteristic domains EGF_CA and GUB_WAK_bind (Fig. 3). The EGF_CA domain is a conserved domain of about forty amino-acid residues found in epidermal growth factor (EGF). This domain is required for calcium-binding which may be crucial for numerous protein-protein interactions [46, 47]. The cysteine-rich GUB_WAK_bind is a unique domain of WAKs, it is responsible for transmitting signals (like pectin fragments) outside cell walls to cells and activating corresponding physiological processes [48].

Gene duplication plays an important role in plant evolution, different models of duplicated gene evolution have been proposed to evaluate selective pressure [49]. Clearly, the WAK/WAKL gene has undergone duplicate during evolution, the branch model showed that WAK/WAKL underwent a strong purifying selection (Table 1). However, the site model revealed that almost all of the positive selection sites are located in N-terminal within first 200aa region, corresponding to the typical domain GUB_WAK_bind (Fig. 3; Table 2). The similar results have been reported in another subfamily of RLK, leucine-rich repeat receptor-like kinases (LRR-RLK) in angiosperms. Four main targets of positive selection amino acids were found in the N-terminal extracellular domain (the leucine-rich repeat domains) [50]. These positive selection sites in LRR have been inferred for genes involved in biotic stress: Xa21 and Xa4, which confer resistance to the bacterial blight disease, was found to have evolved under positive selection in rice [10, 51]; FLS2 in Arabidopsis, involved in responses to multiple biotic stress, shows a signature of rapid fixation of an adaptive allele [52]. GUB_WAK_bind domain plays an important function in recognizing to extracellular signals, mainly oligogalacturonides [53]. Previous studies showed pathogens absorbed nutrients from plants by degrading plant cell walls which will produced oligogalacturonides. At the same time, oligogalacturonides act as a danger signal to induce plant defense response [48, 54, 55]. Interestingly, oligogalacturonide’s biological activity varies with their degree of polymerization (DP) and concentration. For example, oligogalacturonides with 12 DP and 2 ~ 6 DP had the highest activity in soybean [56] and wheat [57], respectively. In general, the dynamics and plasticity of this region in the WAK/WAKL genes implied Rosaceae has a broad tool set to respond to variously environmental challenges, thus undergoes positive selection.

Enrichment of promoter *cis*-element classification of *WAK/WAKL* revealed that Clade II prominently responds to MeJA, one of the major hormones for plant defense response (Table 3). This result was consistent with that 4 of 11 Clade II *WAK/WAKL* genes were reported involved in plant defense (Fig. 2). Furthermore, compared to other clades, Clade IV & V were tended to response to abiotic stress. Through transcriptome analysis of rose petals in response to different hormone treatments, it was found that the response intensity of each subfamily to different hormones was indeed different. For example, it is evident that Clade V has a strong response to ABA as well as Clade I to JA. This may be slightly different from the analysis of promoters, after all the rule of rose does not necessarily represent the universal conclusion of rosaceae crops, but the heat map (Fig. 4) does provide a good resource for study of rose. As WAK/WAKL plays a wide range of biological functions in growth [58], development [59] and interaction with stress factors [60], in further studies of WAK/WAKL genes, we can predict their functions in advance via different clades.

Orthologous group is a group of genes across different crops, such genes originated from a same gene or a same syntenic block from the last common ancestor before the crops diverging [61]. Those orthologous genes have been selectively retained during the respective evolutionary processes, not only because the similarity of amino acid sequences, but also the collinearity relationships formed by the nearby genes in the genome. Therefore, a group of genes with orthologous relationships will perform similar biological function [62]. Here, we identified four orthologous groups throughout four Rosaceae crops. Based on rose-*B. cinerea* interaction study [34], and transient silencing of *RcWAK8* in rose petals exhibited increased susceptibility to *B. cinerea*. We speculated OG1 and OG4 which contained *B. cinerea* induced *RcWAK8* and *RcWAK22* may play a role in resistance to *B. cinerea*, and this hypothesis was verified by qRT-PCR. It is proved that it is feasible to infer the function of orthologous genes in other crops by orthologous group, which provides theoretical reference and basis for exploring the function of WAK/WAKL gene in other Rosaceae plants.

## Conclusion

In conclusion, this study identified 131 WAK/WAKL family members through Rosaceae crops, apples, peaches and strawberries. The number and structure of WAK/WAL family in different crops were analyzed in detail. By comparing the phylogenetic evolution, selective pressure, collinearity relationship of WAK/WAKL throughout four Rosaceae crops, including roses. Finally, one RcWAK (RcWAK8) was verified and three FvWAK genes (FvWAK1, FvWAK5 and FvWAK9) were speculated, which were excavated through the orthologous gene group, were involved to gray mold resistance. This study offers a new idea to infer gene function with the help of the orthogonal group, also provides gene resources for the research of WAK/WAKL gene family, and help understand the function and evolution of Rosaceae WAK/WAKL.

## Electronic supplementary material

Below is the link to the electronic supplementary material.

**Supplementary Data. Supplementary Table1** *Cis*-element categories based on biological process. **Supplementary Table 2** Primers used in the experiment. **Supplementary Table 3** Predicted WAK/WAKL family members in strawberrySupplementary Table 4 Predicted WAK/WAKL family members in apple. **Supplementary Table 5** Predicted WAK/WAKL family members in peach. **Supplementary Table 6** Number of WAK/WAKL genes in different species. **Supplementary Figure 1** Venn diagrams of Genes containing different domains. (a) to (c) represents the quantitative relationships of apple, peach and strawberry respectively. GUB_ WAK_ Bind, galacturonan binding domain, EGF_ CA, calcium binding EGF domain, PKinase, serine/threonine kinase. SignalP&TM, signal peptide and transmembrane helix. **Supplementary Figure 2** DNA structures and conserved domains of the WAK/WAKL gene family. **Supplementary Figure 3** Paralogous genes pairs in four Rosaceae crops. (a) to (d) represent microsyntenic analysis of apple, peach, rose and strawberry, respectively. The grey lines represent pairs of genes that has syntenic relationship around the genome, and the highlighted red line indicating paralogous pairs ofWAK/WAKL family. **Supplementary Figure 4** Syntenic map of orthologous genes running through Rosaceae species. The different coloured bars indicate their chromosomes, the grey lines indicate pairs of genes that are covalently related, and the highlighted red lines mean both of the genes from the WAK/WAKL family. **Supplementary Figure 5** Comparison of CDS (coding sequence) of *RcWAK2* and *RcWAK22*. Sequence alignment was performed by DNAMAN with default parameters in pairwise alignment.

## Data Availability

The datasets used and/or analyzed during the current study has been downloaded from Genome Database for Rosaceae (http://www.rosaceae.org/). The genome data was also available in the following links: gddh13 v1.1 for Apple (*Malus* x *domestica*) (https://iris.angers.inra.fr/gddh13/), Fragaria vesca v4.0.a1 for strawberry (*Fragaria vesca*) (https://github.com/wurmlab/flo), Prunus persica v2.0 for peach (*Prunus persica*) (https://phytozome-next.jgi.doe.gov/info/Ppersica_v2_1). The plant materials are available from the corresponding author on reasonable request.
