# Supplementary material for: Comparative genomics analysis of WAK/WAKL family in Rosaceae identify candidate WAKs involved in the resistance to Botrytis cinerea
Source: BMC Genomics. 2023 Jun 19;24:337. doi: 10.1186/s12864-023-09371-9 (PMC10278292; doi:10.1186/s12864-023-09371-9)
Supplement: Supplementary file 1 — Supplementary Data. Supplementary Table1 Cis-element categories based on biological process. Supplementary Table 2 Primers used in the experiment. Supplementary Table 3 Predicted WAK/WAKL family members in strawberrySupplementary Table 4 Predicted WAK/WAKL family members in apple. Supplementary Table 5 Predicted WAK/WAKL family members in peach. Supplementary Table 6 Number of WAK/WAKL genes in different species. Supplementary Figure 1 Venn diagrams of Genes containing different domains. (a) to (c) represents the quantitative relationships of apple, peach and strawberry respectively. GUB_ WAK_ Bind, galacturonan binding domain, EGF_ CA, calcium binding EGF domain, PKinase, serine/threonine kinase. SignalP&TM, signal peptide and transmembrane helix. Supplementary Figure 2 DNA structures and conserved domains of the WAK/WAKL gene family. Supplementary Figure 3 Paralogous genes pairs in four Rosaceae crops. (a) to (d) represent microsyntenic analysis of apple, peach, rose and strawberry, respectively. The grey lines represent pairs of genes that has syntenic relationship around the genome, and the highlighted red line indicating paralogous pairs ofWAK/WAKL family. Supplementary Figure 4 Syntenic map of orthologous genes running through Rosaceae species. The different coloured bars indicate their chromosomes, the grey lines indicate pairs of genes that are covalently related, and the highlighted red lines mean both of the genes from the WAK/WAKL family. Supplementary Figure 5 Comparison of CDS (coding sequence) of RcWAK2 and RcWAK22. Sequence alignment was performed by DNAMAN with default parameters in pairwise alignment. [file 12864_2023_9371_MOESM1_ESM.docx]

# Supplementary Data

**Supplementary Table 1 *Cis*-element categories based on biological process**

| **Major categories** | **Description** | **Motifs** |
| --- | --- | --- |
| Plant development and growth | Cell cycle regulation | MSA-like |
|  | Crcadian control | circadian |
|  | Endosperm expression | AACA_motif |
|  |  | GCN4_motif |
|  | Meristem expression | CAT-box |
|  |  | NON-box |
|  | Palisade mesophyll cells differentiation | HD-Zip 1 |
|  | Seed-specific regulation | RY-element |
| Phytohormone response | Abscisic acid response | ABRE |
|  | Auxin response | TGA-element |
|  |  | AuxRR-core |
|  |  | AuxRE |
|  |  | GATA-box |
|  |  | TGA-box |
|  | Gibberellin response | GARE-motif |
|  |  | P-box |
|  |  | TATC-box |
|  | Jasmonic acid response | TGACG-motif |
|  |  | CGTCA-motif |
|  | Salicylic acid response | TCA-element |
| Abiotic stress response | Anaerobic Induction | ARE |
|  |  | GC-motif |
|  | Drought Induction | MBS |
|  | Low-temperature responsive | LTR |
|  | Wound-responsive element | WUN-motif |

**Supplementary Table 2 Primers used in the experiment**

| **Primer name** | **Sequence (5’-3’)** | |
| --- | --- | --- |
| FvWAK1 (for qRT-PCR) | | F: TGGGAGATGATTATGTGATTGTTTT |
|  |  | R: ACACTTCCTATCCTAGCATTGCTTT |
| FvWAK2 (for qRT-PCR) | | F: GTACGTGGAACATTTGGTTATTTG |
|  |  | R: TCTACTTTCTTGCGACCTTATCAT |
| FvWAK5 (for qRT-PCR) | | F: CCGCAAAAAACAATCAAAGACGACG |
|  |  | R: AACGAACAGAACCAAGAAGCCCCCC |
| FvWAK8 (for qRT-PCR) | | F: GCCGATTATCAATCTTCATCTGTGTAT |
|  |  | R: CTAATTGCTAACCGTTTTCTTCTAGGA |
| FvWAK9 (for qRT-PCR) | | F: GGCCTACCATGAAAGAAGTAGCGA |
|  |  | R: AAGCATTTGAAGGTGACGGAGCAA |
| FvACTIN (for qRT-PCR) | | F: GCCAACCGTGAGAAGATG |
|  |  | R: TCCAGAGTCAAGAACAATACCAG |
| RcWAK8 (for qRT-PCR) | | F: GTGAAATGTAAGTTAATGTGACCCTGC |
|  |  | R: GCCTTCTTGCTTTTGCACCAAGTACAA |
| RcUBI (for qRT-PCR) | | F: CACAAGCACGCAAACCCTAT |
|  |  | R: GGAGCATGAGCCAAATGGAG |
| RcWAK8(for VIGS) | | F: TCCAAGCAGTGGTGGACGCAGAAAA |
|  |  | R: AAAAGCTTACTGATAGCCTGATACT |

**Supplementary Table 3 Predicted WAK/WAKL family members in strawberry**

| **Gene** | **Accession number ^a^** | **Chr.^b^** | **Position ^c^** | **Exon** | **Intron** | **CDS (bp)** | **Amino Acids** | **Clade** |
| --- | --- | --- | --- | --- | --- | --- | --- | --- |
| FvWAK1 | FvH4_2g35060.1 | 2 | 25.91765 | 6 | 5 | 4793 | 1450 | Ⅱ |
| FvWAK2 | FvH4_3g09860.1 | 3 | 5.742648 | 3 | 2 | 2283 | 760 | Ⅲ |
| FvWAK3 | FvH4_3g10350.1 | 3 | 6.054429 | 3 | 2 | 2752 | 787 | Ⅰ |
| FvWAK4 | FvH4_3g10360.1 | 3 | 6.058956 | 3 | 2 | 2287 | 754 | Ⅰ |
| FvWAK5 | FvH4_3g10510.1 | 3 | 6.189099 | 3 | 2 | 1773 | 570 | Ⅰ |
| FvWAK6 | FvH4_3g10610.1 | 3 | 6.320654 | 3 | 2 | 2307 | 768 | Ⅰ |
| FvWAK7 | FvH4_3g10620.1 | 3 | 6.324417 | 7 | 6 | 5111 | 788 | Ⅰ |
| FvWAK8 | FvH4_3g10690.1 | 3 | 6.36195 | 7 | 6 | 4796 | 1545 | Ⅰ |
| FvWAK9 | FvH4_3g11730.1 | 3 | 6.923924 | 3 | 2 | 2792 | 778 | Ⅰ |
| FvWAK10 | FvH4_5g06860.1 | 5 | 4.028769 | 3 | 2 | 2458 | 752 | Ⅲ |
| FvWAK11 | FvH4_5g06940.1 | 5 | 4.110458 | 5 | 4 | 2209 | 705 | Ⅲ |
| FvWAK12 | FvH4_6g53140.1 | 6 | 39.0102 | 7 | 6 | 7019 | 2266 | Ⅱ |
| FvWAK13 | FvH4_7g31880.1 | 7 | 22.99794 | 3 | 2 | 2400 | 799 | Ⅰ |
| FvWAK14 | FvH4_7g31920.1 | 7 | 23.01787 | 3 | 2 | 3031 | 797 | Ⅰ |
| FvWAKL1 | FvH4_1g03330.1 | 1 | 1.84565 | 6 | 5 | 4072 | 607 | Ⅴ |
| FvWAKL2 | FvH4_1g07120.1 | 1 | 3.764624 | 9 | 8 | 3120 | 925 | Ⅴ |
| FvWAKL3 | FvH4_2g12540.1 | 2 | 11.01504 | 6 | 5 | 4253 | 1011 | Ⅳ |
| FvWAKL4 | FvH4_3g24040.1 | 3 | 17.08778 | 2 | 1 | 1977 | 658 | Ⅲ |
| FvWAKL5 | FvH4_3g35290.1 | 3 | 30.42648 | 3 | 2 | 2661 | 736 | Ⅲ |
| FvWAKL6 | FvH4_4g34420.1 | 4 | 32.21677 | 3 | 2 | 3294 | 601 | Ⅴ |
| FvWAKL7 | FvH4_5g11780.1 | 5 | 6.668576 | 4 | 3 | 2703 | 651 | Ⅴ |
| FvWAKL8 | FvH4_5g22270.1 | 5 | 13.67487 | 3 | 2 | 1991 | 574 | Ⅴ |
| FvWAKL9 | FvH4_6g23830.1 | 6 | 17.91204 | 7 | 6 | 3690 | 644 | Ⅴ |
| FvWAKL10 | FvH4_6g37660.1 | 6 | 29.64871 | 3 | 2 | 2286 | 733 | Ⅲ |
| FvWAKL11 | FvH4_6g37670.1 | 6 | 29.67993 | 3 | 2 | 2399 | 745 | Ⅲ |
| FvWAKL12 | FvH4_6g38990.1 | 6 | 30.82468 | 7 | 6 | 5869 | 756 | Ⅲ |
| FvWAKL13 | FvH4_6g39000.1 | 6 | 30.8359 | 3 | 2 | 2133 | 710 | Ⅲ |
| FvWAKL14 | FvH4_6g39020.1 | 6 | 30.84541 | 3 | 2 | 2223 | 740 | Ⅲ |
| FvWAKL15 | FvH4_7g04160.1 | 7 | 4.791193 | 2 | 1 | 1851 | 616 | Ⅴ |
| FvWAKL16 | FvH4_7g05990.1 | 7 | 6.183596 | 7 | 6 | 2721 | 674 | Ⅴ |
| FvWAKL17 | FvH4_7g06010.1 | 7 | 6.200855 | 9 | 8 | 3296 | 1039 | Ⅴ |
| FvWAKL18 | FvH4_7g06020.1 | 7 | 6.207926 | 3 | 2 | 2076 | 623 | Ⅴ |
| FvWAKL19 | FvH4_7g06040.1 | 7 | 6.2254 | 6 | 5 | 2051 | 590 | Ⅴ |
| FvWAKL20 | FvH4_7g17870.1 | 7 | 15.05287 | 3 | 2 | 1970 | 636 | Ⅳ |
| FvWAKL21 | FvH4_7g20630.1 | 7 | 16.7249 | 5 | 4 | 2011 | 586 | Ⅴ |
| FvWAKL22 | FvH4_7g23160.1 | 7 | 18.12545 | 3 | 2 | 2385 | 627 | Ⅳ |

a Available at https://lipm-browsers.toulouse.inra.fr/pub/RchiOBHm-V2/.
b Chromosome.
c Starting position (Mb).

**Supplementary Table 4 Predicted WAK/WAKL family members in apple**

| **Gene** | **Accession number ^a^** | **Chr.^b^** | **Position ^c^** | **Exon** | **Intron** | **CDS (bp)** | **Amino Acids** | **Clade** |
| --- | --- | --- | --- | --- | --- | --- | --- | --- |
| MdWAK1 | MD05G1269900 | 5 | 40.48713 | 3 | 2 | 2265 | 754 | Ⅰ |
| MdWAK2 | MD08G1107600 | 8 | 9.459183 | 4 | 3 | 2198 | 714 | Ⅱ |
| MdWAK3 | MD08G1107900 | 8 | 9.481554 | 3 | 2 | 2339 | 772 | Ⅲ |
| MdWAK4 | MD10G1248500 | 10 | 34.16426 | 5 | 4 | 2679 | 892 | Ⅰ |
| MdWAK5 | MD10G1250500 | 10 | 34.29704 | 5 | 4 | 2142 | 713 | Ⅰ |
| MdWAK6 | MD10G1251200 | 10 | 34.45409 | 3 | 2 | 2545 | 766 | Ⅰ |
| MdWAK7 | MD10G1251400 | 10 | 34.46982 | 3 | 2 | 2286 | 761 | Ⅰ |
| MdWAK8 | MD13G1004600 | 13 | 0.280748 | 3 | 2 | 2283 | 760 | Ⅱ |
| MdWAK9 | MD15G1088400 | 15 | 6.166287 | 3 | 2 | 2343 | 780 | Ⅱ |
| MdWAKL1 | MD01G1059700 | 1 | 16.35682 | 6 | 5 | 2404 | 695 | Ⅴ |
| MdWAKL2 | MD01G1091100 | 1 | 20.62039 | 3 | 2 | 2052 | 683 | Ⅳ |
| MdWAKL3 | MD01G1139800 | 1 | 24.93618 | 3 | 2 | 2321 | 621 | Ⅳ |
| MdWAKL4 | MD02G1247100 | 2 | 29.73918 | 6 | 5 | 1897 | 617 | Ⅴ |
| MdWAKL5 | MD02G1249300 | 2 | 29.98542 | 6 | 5 | 1959 | 652 | Ⅴ |
| MdWAKL6 | MD02G1273500 | 2 | 32.80932 | 3 | 2 | 2307 | 670 | Ⅴ |
| MdWAKL7 | MD02G1273700 | 2 | 32.82504 | 3 | 2 | 2086 | 604 | Ⅴ |
| MdWAKL8 | MD02G1274000 | 2 | 32.85803 | 4 | 3 | 2259 | 631 | Ⅴ |
| MdWAKL9 | MD02G1274600 | 2 | 32.9126 | 3 | 2 | 2002 | 613 | Ⅴ |
| MdWAKL10 | MD04G1233200 | 4 | 31.22011 | 2 | 1 | 2738 | 635 | Ⅴ |
| MdWAKL11 | MD06G1218900 | 6 | 35.15848 | 4 | 3 | 2401 | 652 | Ⅳ |
| MdWAKL12 | MD07G1070400 | 7 | 6.662745 | 6 | 5 | 2013 | 670 | Ⅴ |
| MdWAKL13 | MD07G1070700 | 7 | 6.679087 | 7 | 6 | 2395 | 671 | Ⅴ |
| MdWAKL14 | MD07G1161300 | 7 | 23.64085 | 3 | 2 | 1938 | 645 | Ⅳ |
| MdWAKL15 | MD09G1145200 | 9 | 11.28813 | 3 | 2 | 2295 | 764 | Ⅲ |
| MdWAKL16 | MD09G1145300 | 9 | 11.29893 | 3 | 2 | 2178 | 725 | Ⅲ |
| MdWAKL17 | MD09G1145500 | 9 | 11.31585 | 4 | 3 | 2962 | 737 | Ⅲ |
| MdWAKL18 | MD12G1149600 | 12 | 22.97696 | 2 | 1 | 1863 | 620 | Ⅳ |
| MdWAKL19 | MD12G1250800 | 12 | 32.08086 | 2 | 1 | 2109 | 702 | Ⅴ |
| MdWAKL20 | MD12G1251100 | 12 | 32.09172 | 4 | 3 | 1947 | 647 | Ⅴ |
| MdWAKL21 | MD12G1251200 | 12 | 32.0945 | 2 | 1 | 1932 | 643 | Ⅴ |
| MdWAKL22 | MD13G1036100 | 13 | 2.51644 | 5 | 4 | 1524 | 500 | Ⅳ |
| MdWAKL23 | MD13G1037900 | 13 | 2.586111 | 3 | 2 | 1965 | 650 | Ⅴ |
| MdWAKL24 | MD14G1096100 | 14 | 14.57906 | 1 | 0 | 1902 | 633 | Ⅳ |
| MdWAKL25 | MD14G1228700 | 14 | 31.02785 | 4 | 3 | 2078 | 682 | Ⅳ |
| MdWAKL26 | MD17G1131600 | 17 | 11.60009 | 3 | 2 | 2178 | 725 | Ⅲ |

a Available at https://lipm-browsers.toulouse.inra.fr/pub/RchiOBHm-V2/.
b Chromosome.
c Starting position (Mb).

**Supplementary Table 5 Predicted WAK/WAKL family members in peach**

| **Gene** | **Accession number ^a^** | **Chr.^b^** | **Position ^c^** | **Exon** | **Intron** | **CDS (bp)** | **Amino Acids** | **Clade** |
| --- | --- | --- | --- | --- | --- | --- | --- | --- |
| PpWAK1 | Prupe.1G442500.1 | 1 | 37.54409 | 3 | 2 | 2301 | 766 | Ⅱ |
| PpWAK2 | Prupe.4G091900.1 | 4 | 4.58662 | 3 | 2 | 2265 | 754 | Ⅰ |
| PpWAK3 | Prupe.4G092700.1 | 4 | 4.651056 | 3 | 2 | 2226 | 741 | Ⅰ |
| PpWAK4 | Prupe.4G092900.1 | 4 | 4.672275 | 3 | 2 | 2199 | 732 | Ⅰ |
| PpWAK5 | Prupe.4G093100.1 | 4 | 4.690237 | 3 | 2 | 2361 | 786 | Ⅰ |
| PpWAK6 | Prupe.4G093100.2 | 4 | 4.690366 | 3 | 2 | 2325 | 774 | Ⅰ |
| PpWAK7 | Prupe.4G093200.1 | 4 | 4.697706 | 3 | 2 | 2259 | 752 | Ⅰ |
| PpWAK8 | Prupe.4G093300.1 | 4 | 4.70104 | 3 | 2 | 2259 | 752 | Ⅰ |
| PpWAK9 | Prupe.4G093400.1 | 4 | 4.705881 | 3 | 2 | 2247 | 748 | Ⅰ |
| PpWAK10 | Prupe.4G093500.1 | 4 | 4.709207 | 3 | 2 | 2283 | 760 | Ⅰ |
| PpWAK11 | Prupe.4G094100.1 | 4 | 4.741372 | 4 | 3 | 2259 | 752 | Ⅰ |
| PpWAK12 | Prupe.4G094500.1 | 4 | 4.766987 | 4 | 3 | 2337 | 778 | Ⅰ |
| PpWAKL1 | Prupe.1G315000.1 | 1 | 30.42904 | 3 | 2 | 1902 | 633 | Ⅴ |
| PpWAKL2 | Prupe.1G315000.2 | 1 | 30.4291 | 2 | 1 | 1869 | 622 | Ⅴ |
| PpWAKL3 | Prupe.1G315900.1 | 1 | 30.48858 | 4 | 3 | 2037 | 678 | Ⅳ |
| PpWAKL4 | Prupe.1G315900.2 | 1 | 30.4886 | 3 | 2 | 2007 | 668 | Ⅳ |
| PpWAKL5 | Prupe.1G318900.1 | 1 | 30.63126 | 6 | 5 | 1227 | 408 | Ⅴ |
| PpWAKL6 | Prupe.2G047400.1 | 2 | 5.472545 | 3 | 2 | 1890 | 629 | Ⅴ |
| PpWAKL7 | Prupe.2G047400.2 | 2 | 5.472365 | 3 | 2 | 1857 | 618 | Ⅴ |
| PpWAKL8 | Prupe.2G047500.1 | 2 | 5.475758 | 4 | 3 | 1962 | 653 | Ⅴ |
| PpWAKL9 | Prupe.2G087100.1 | 2 | 13.75632 | 6 | 5 | 1995 | 664 | Ⅴ |
| PpWAKL10 | Prupe.2G087800.1 | 2 | 13.8753 | 6 | 5 | 2064 | 687 | Ⅴ |
| PpWAKL11 | Prupe.2G088100.1 | 2 | 13.91298 | 6 | 5 | 1983 | 660 | Ⅴ |
| PpWAKL12 | Prupe.2G088900.1 | 2 | 14.03523 | 3 | 2 | 1812 | 603 | Ⅴ |
| PpWAKL13 | Prupe.2G089000.2 | 2 | 14.05951 | 3 | 2 | 1797 | 598 | Ⅴ |
| PpWAKL14 | Prupe.2G093400.1 | 2 | 14.81271 | 4 | 3 | 1644 | 547 | Ⅴ |
| PpWAKL15 | Prupe.2G103300.1 | 2 | 15.90722 | 4 | 3 | 2121 | 706 | Ⅴ |
| PpWAKL16 | Prupe.2G200600.1 | 2 | 23.751 | 3 | 2 | 1935 | 644 | Ⅳ |
| PpWAKL17 | Prupe.2G240400.1 | 2 | 26.02442 | 3 | 2 | 1890 | 629 | Ⅳ |
| PpWAKL18 | Prupe.3G145600.1 | 3 | 15.89489 | 5 | 4 | 1398 | 465 | Ⅴ |
| PpWAKL19 | Prupe.3G145700.1 | 3 | 15.89912 | 3 | 2 | 1980 | 659 | Ⅴ |
| PpWAKL20 | Prupe.3G145800.1 | 3 | 15.91152 | 3 | 2 | 2151 | 716 | Ⅴ |
| PpWAKL21 | Prupe.3G146200.1 | 3 | 15.99703 | 3 | 2 | 1917 | 638 | Ⅴ |
| PpWAKL22 | Prupe.3G146500.1 | 3 | 16.02174 | 4 | 3 | 2046 | 681 | Ⅳ |
| PpWAKL23 | Prupe.3G180500.1 | 3 | 19.63502 | 3 | 2 | 2205 | 734 | Ⅲ |
| PpWAKL24 | Prupe.3G180600.1 | 3 | 19.63842 | 3 | 2 | 2283 | 760 | Ⅲ |
| PpWAKL25 | Prupe.3G181100.1 | 3 | 19.68919 | 3 | 2 | 2244 | 747 | Ⅲ |
| PpWAKL26 | Prupe.3G181100.2 | 3 | 19.68919 | 2 | 1 | 2127 | 708 | Ⅲ |
| PpWAKL27 | Prupe.3G181200.1 | 3 | 19.69577 | 3 | 2 | 2175 | 724 | Ⅲ |
| PpWAKL28 | Prupe.3G181200.2 | 3 | 19.69578 | 4 | 3 | 2175 | 724 | Ⅲ |
| PpWAKL29 | Prupe.3G181400.1 | 3 | 19.72138 | 3 | 2 | 1800 | 599 | Ⅲ |
| PpWAKL30 | Prupe.3G181800.1 | 3 | 19.77873 | 3 | 2 | 2256 | 751 | Ⅲ |
| PpWAKL31 | Prupe.3G181900.1 | 3 | 19.7823 | 3 | 2 | 2346 | 781 | Ⅲ |
| PpWAKL32 | Prupe.3G182400.1 | 3 | 19.80251 | 3 | 2 | 2325 | 774 | Ⅲ |
| PpWAKL33 | Prupe.3G182700.1 | 3 | 19.84072 | 3 | 2 | 2301 | 766 | Ⅲ |
| PpWAKL34 | Prupe.4G093000.1 | 4 | 4.678991 | 2 | 1 | 1959 | 652 | Ⅰ |
| PpWAKL35 | Prupe.4G094000.1 | 4 | 4.73672 | 7 | 6 | 1722 | 573 | Ⅰ |
| PpWAKL36 | Prupe.5G051200.1 | 5 | 5.498674 | 3 | 2 | 2271 | 756 | Ⅲ |
| PpWAKL37 | Prupe.5G051600.1 | 5 | 5.570567 | 3 | 2 | 2304 | 767 | Ⅲ |
| PpWAKL38 | Prupe.5G223900.2 | 5 | 17.35145 | 3 | 2 | 1983 | 660 | Ⅳ |
| PpWAKL39 | Prupe.5G223900.3 | 5 | 17.35145 | 4 | 3 | 2019 | 672 | Ⅳ |
| PpWAKL40 | Prupe.6G137900.1 | 6 | 11.19002 | 3 | 2 | 1689 | 562 | Ⅴ |
| PpWAKL41 | Prupe.6G138000.1 | 6 | 11.19971 | 5 | 4 | 2004 | 667 | Ⅴ |
| PpWAKL42 | Prupe.6G142200.1 | 6 | 11.53081 | 3 | 2 | 1737 | 578 | Ⅴ |
| PpWAKL43 | Prupe.6G142300.1 | 6 | 11.53337 | 4 | 3 | 2103 | 700 | Ⅴ |
| PpWAKL44 | Prupe.6G142300.2 | 6 | 11.53337 | 5 | 4 | 1815 | 604 | Ⅴ |
| PpWAKL45 | Prupe.6G261100.1 | 6 | 25.14845 | 3 | 2 | 1926 | 641 | Ⅳ |
| PpWAKL46 | Prupe.6G352900.1 | 6 | 30.03837 | 2 | 1 | 2058 | 685 | Ⅴ |
| PpWAKL47 | Prupe.6G352900.2 | 6 | 30.03851 | 2 | 1 | 2052 | 683 | Ⅴ |
| PpWAKL48 | Prupe.6G358300.1 | 6 | 30.33538 | 2 | 1 | 2133 | 710 | Ⅱ |

a Available at https://lipm-browsers.toulouse.inra.fr/pub/RchiOBHm-V2/.
b Chromosome.
c Starting position

**Supplementary Table 6 Number of WAK/WAKL genes in different species**

| **species** | **Genome_size (Mb)** | **WAK** | **WAKL** | **Total** |
| --- | --- | --- | --- | --- |
| Strawberry (*Fragaria vesca*) | 240 | 14 | 22 | 36 |
| Apple (*Malus domestica*) | 742 | 9 | 26 | 35 |
| Peach (*Prunus persica*) | 265 | 12 | 48 | 60 |
| Rose (*Rosa chinensis*) | 560 | 23 | 45 | 68 |
| Arabidopsis（*Arabidopsis thaliana*） | 125 | 5 | 22 | 27 |
| Rice.japonica (*Oryza sativa subsp. japonica*) | 430 | NA | NA | 130 |
| Rice.indica (*Oryza sativa subsp. indica*) | 430 | NA | NA | 111 |
| Tomato (*Solanum lycopersicum*) | 900 | 11 | 18 | 29 |
| Barley (*Hordeum vulgare*) | 4980 | NA | NA | 91 |
| Cotton.arboreum (*Gossypium arboreum*) | 1746 | 16 | 42 | 58 |
| Cotton. Raimondii (*Gossypium raimondii*) | 880 | 11 | 55 | 66 |
| Cotton. Hirsutum (*Gossypium hirsutum*) | 2340 | 23 | 76 | 99 |
| Walnut (*Juglans regia*) | 606 | 27 | 14 | 41 |


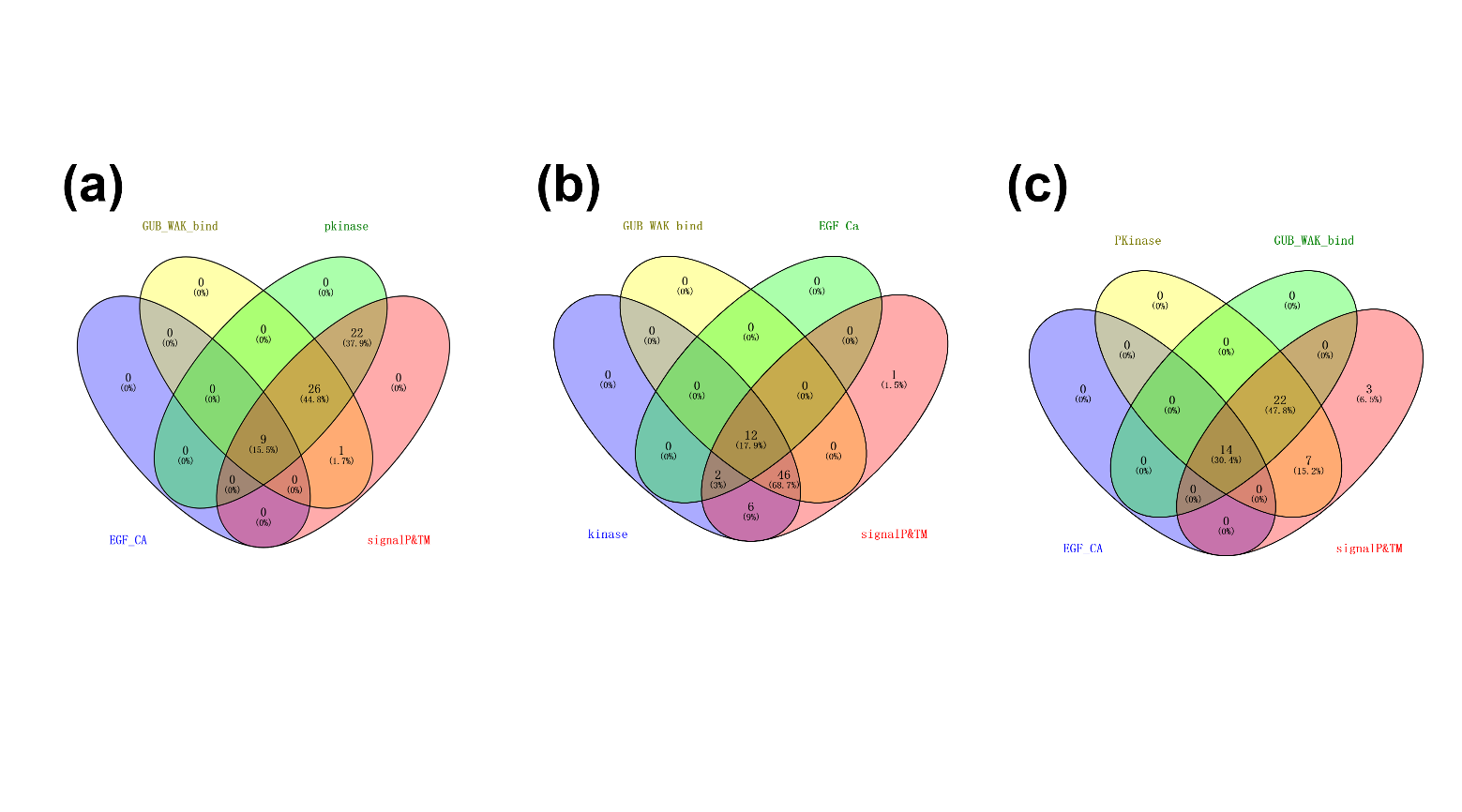


**Supplementary Figure 1 Venn diagrams of Genes containing different domains.** **(a) – (c)** represents the quantitative relationships of apple, peach and strawberry respectively. GUB_ WAK_ Bind, galacturonan binding domain, EGF_ CA, calcium binding EGF domain, PKinase, serine/threonine kinase. SignalP&TM, signal peptide and transmembrane helix.


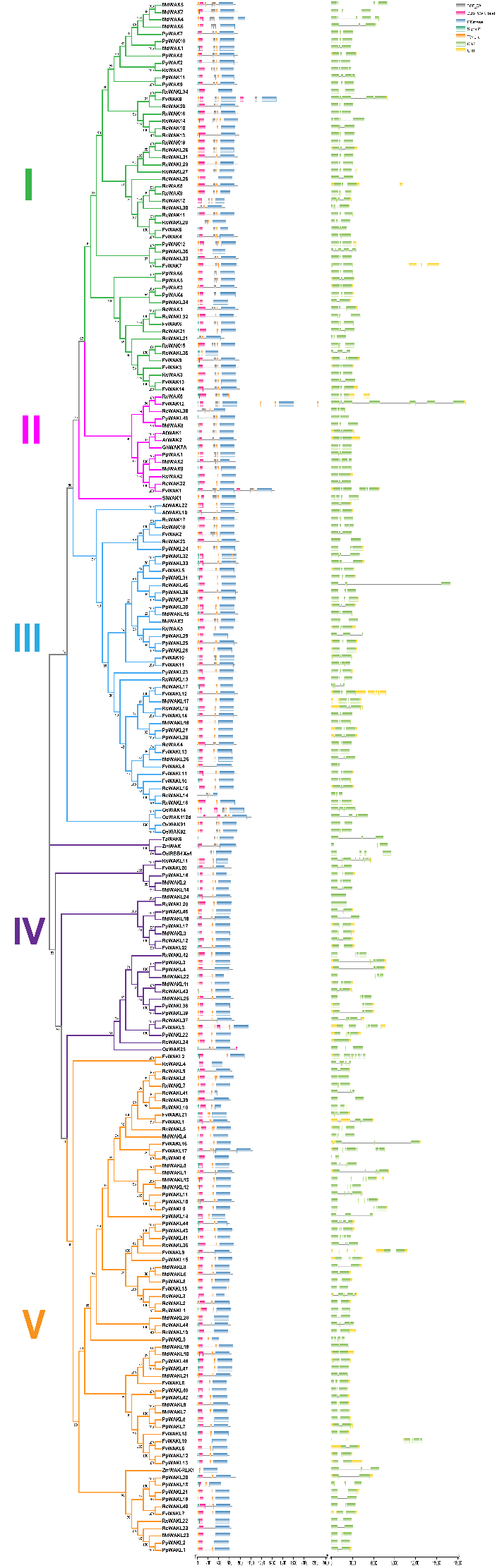


**Supplementary Figure 2 DNA structures and conserved domains of the WAK/WAKL gene family.**

**
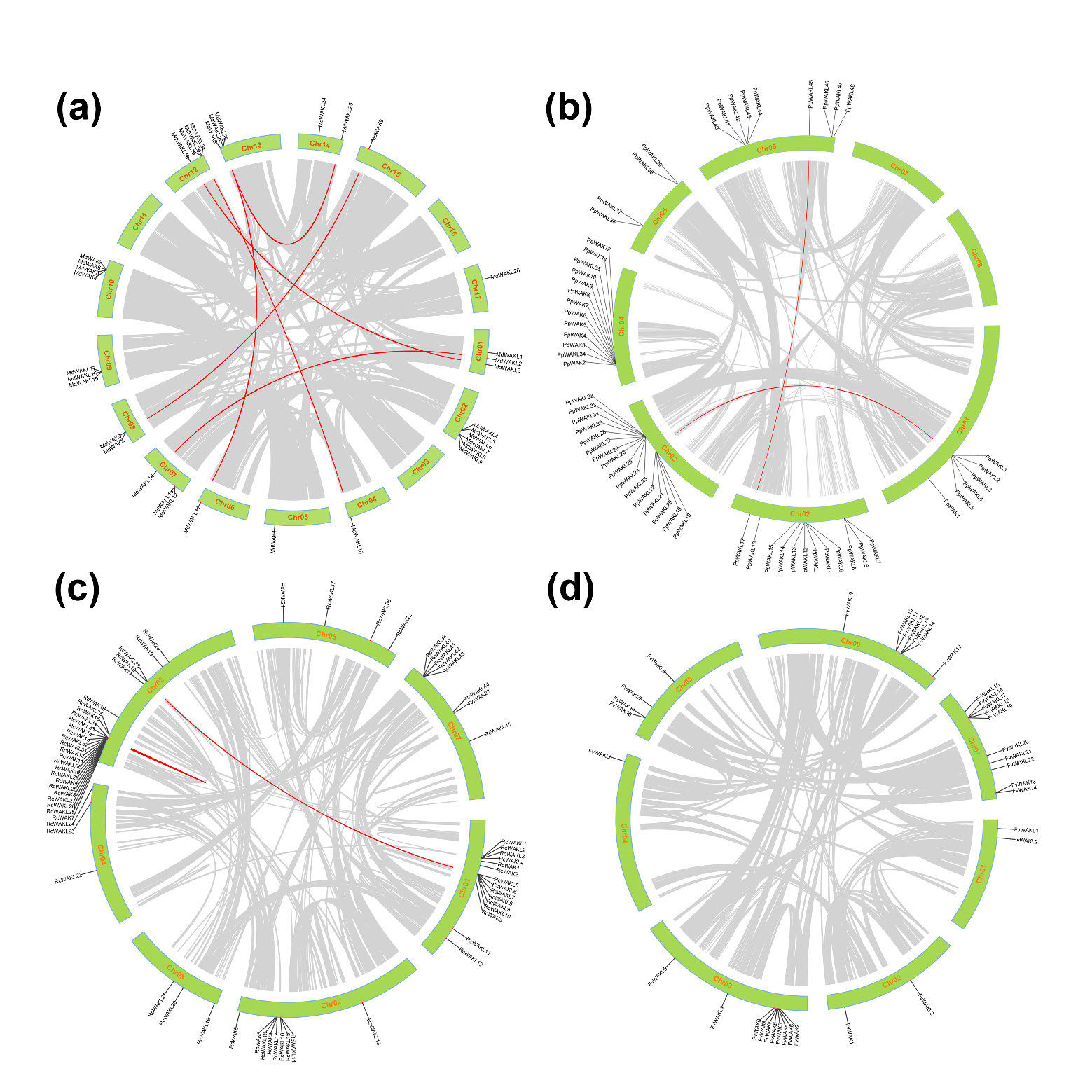
**

**Supplementary Figure 3** **Paralogous genes pairs in four Rosaceae crops. (a) to (d)** represent microsyntenic analysis of apple, peach, rose and strawberry, respectively. The grey lines represent pairs of genes that has syntenic relationship around the genome, and the highlighted red line indicating paralogous pairs ofWAK/WAKL family.


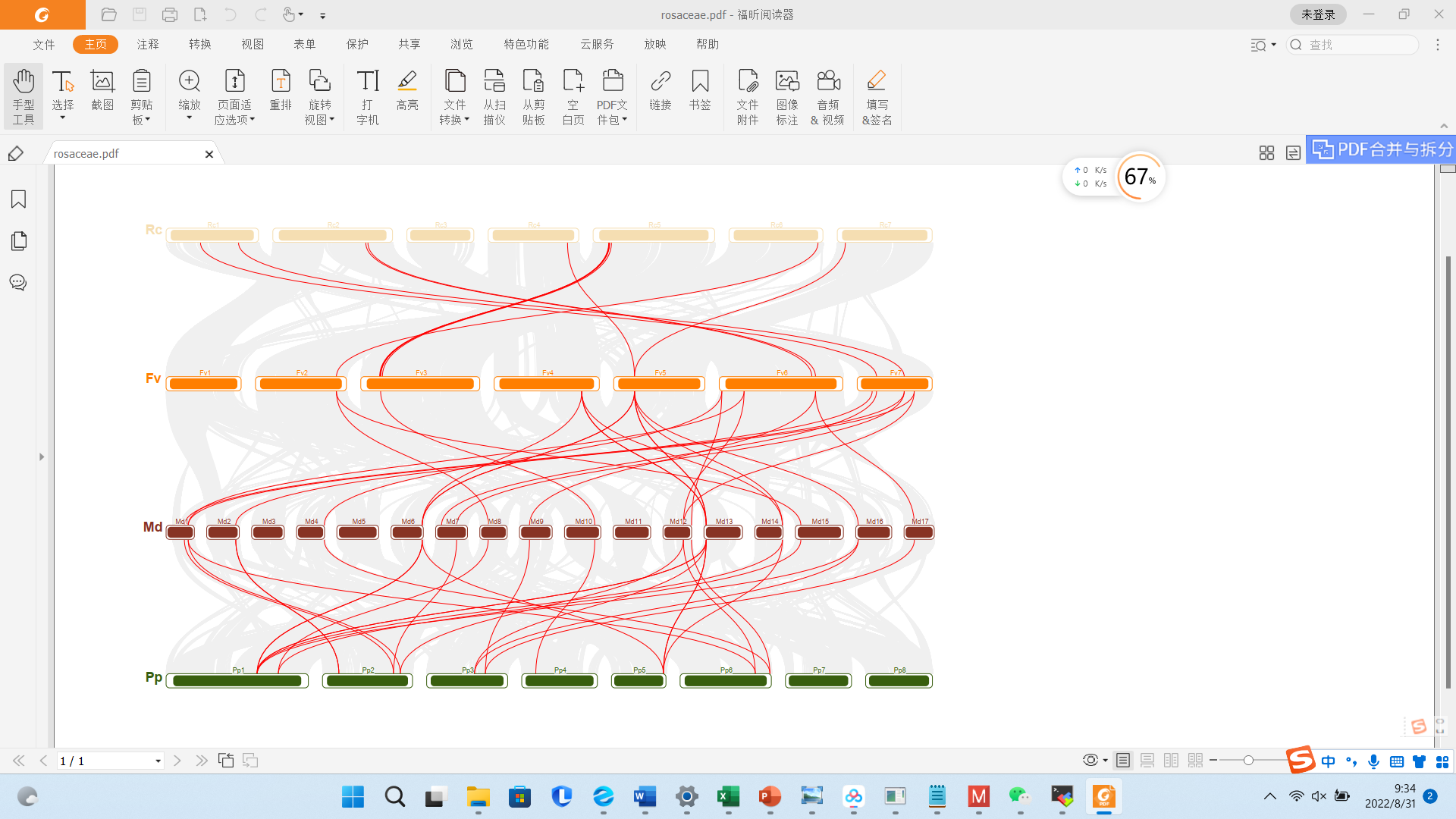


**Supplementary Figure 4 Syntenic map of orthologous genes running through Rosaceae species.** The different coloured bars indicate their chromosomes, the grey lines indicate pairs of genes that are covalently related, and the highlighted red lines mean both of the genes from the WAK/WAKL family.


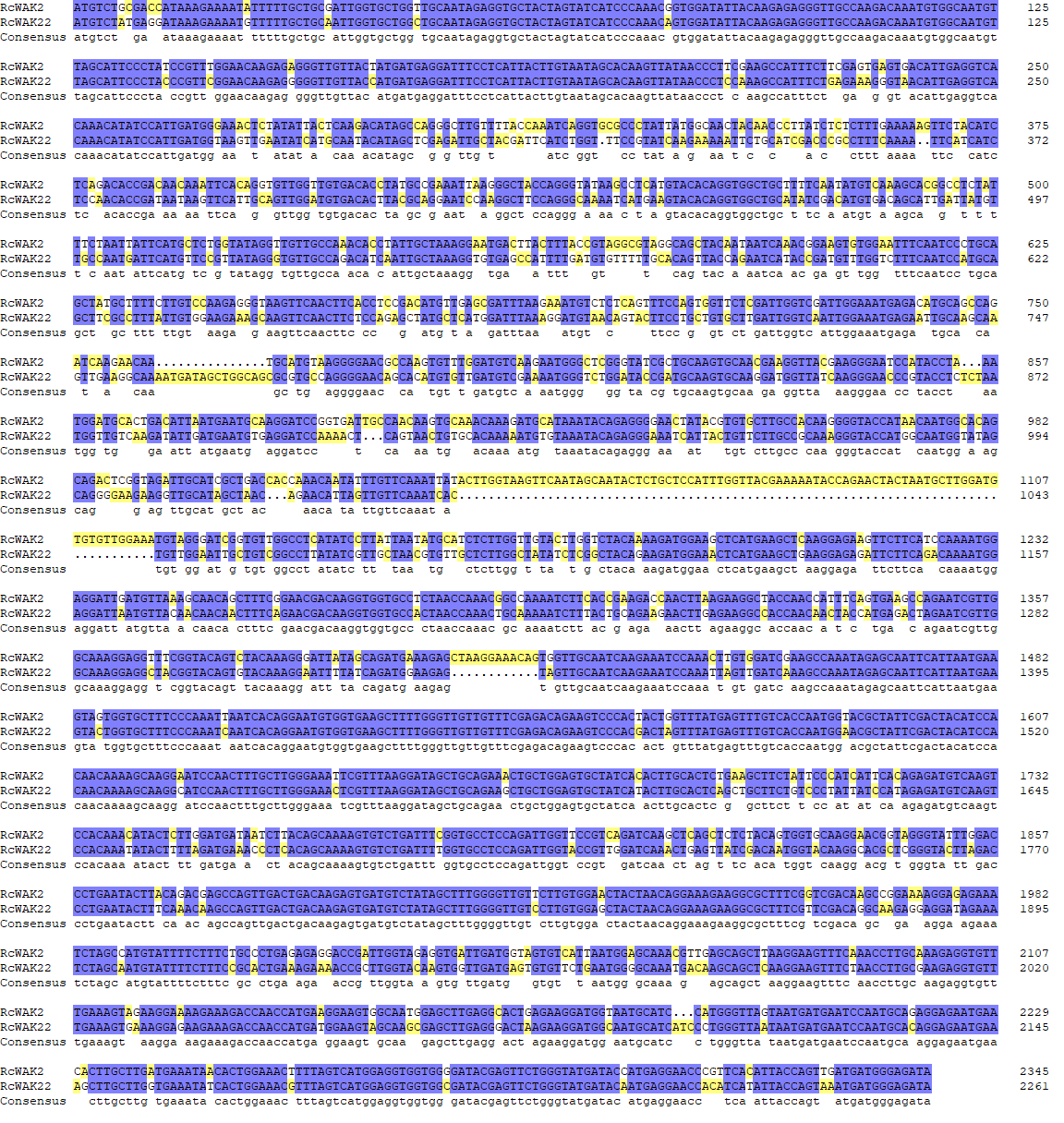


**Supplementary Figure 5 Comparison of CDS (coding sequence) of RcWAK2 and RcWAK22.** Sequence alignment was performed by DNAMAN with default parameters in pairwise alignment.
